# Osthole inhibits bone metastasis of breast cancer

**DOI:** 10.18632/oncotarget.17024

**Published:** 2017-04-11

**Authors:** Chunyu Wu, Zhenping Sun, Baofeng Guo, Yiyi Ye, Xianghui Han, Yuenong Qin, Sheng Liu

**Affiliations:** ^1^ Department of Breast Surgery (Integrated Traditional and Western Medicine), Longhua Hospital, Shanghai University of Traditional Chinese Medicine, Shanghai 200032, China; ^2^ Pharmacology Laboratory of Traditional Chinese Medicine, Longhua Hospital, Shanghai University of Traditional Chinese Medicine, Shanghai 200032, China

**Keywords:** osthole, *Cnidium monnieri (L.) Cusson*, coumarin, breast cancer, bone metastasis

## Abstract

Bone is one of the most common sites for breast cancer metastasis, which greatly contributes to patient morbidity and mortality. Osthole, a major extract from *Cnidium monnieri (L.)*, exhibits many biological and pharmacological activities, however, its potential as a therapeutic agent in the treatment of breast cancer bone metastases remain poorly understood. In this study, we set out to investigate whether osthole could inhibit breast cancer metastasis to bone in mice and clarified the potential mechanism of this inhibition. In the murine model of breast cancer osseous metastasis, mice that received osthole developed significantly less bone metastases and displayed decreased tumor burden when compared with mice in the control group. Osthole inhibited breast cancer cell growth, migration, and invasion, and induced apoptosis of breast cancer cells. Additionally, it also regulated OPG/RANKL signals in the interactions between bone cells (osteoblasts and osteoclasts) and cancer cells. Besides, it also inhibited TGF-β/Smads signaling in breast cancer metastasis to bone in MDA-231BO cells. The results of this study suggest that osthole has real potential as a therapeutic candidate in the treatment of breast cancer patients with bone metastases.

## INTRODUCTION

In China, the plant *Cnidium monnieri (L.) Cusson* has been used medicinally for centuries. The dried fruits of *C. monnieri* have been used widely in Chinese herbal prescriptions for the treatment of such conditions as itchy skin and eczema, pain in female genitalia, sexual dysfunction, and to promote bone regulation [1–4]. In traditional Chinese medicinal, *C. monnieri* is found in many formulations, such as Bushen Zhuanggu, BuGuZhi Wan, Wenshen Zhuanggu, and FaZhi Heidou. In particular, the Bushen Zhuanggu formula has been clinically prescribed for many years as an alternative therapy for the treatment of metastatic breast cancer. In a retrospective cohort study, the Bushen Zhuanggu formula was used clinically as an adjuvant in breast cancer patients with bone metastases. Patients receiving the formula showed improvements in bone pain, reduction in the incidence of bone-related events, and a demonstrated delay in and decreased number of osteolytic lesions. Our previous studies have also documented that, in mice, a Chinese formula including *P. corylifolia-C. monnieri* inhibits bone metastasis in breast cancer, possibly due to alterations in the OPG/RANKL/RANK system [5, 6].

Osthole (7-methoxy-8-isopentenoxycoumarin) is a coumarin-derivative extract of *C. monnieri* that has been shown to inhibit many pathological disorders, such as allergies, inflammation, HIV activity, diabetes, as well as provide protective effects for the liver [7–12]. It can also improve learning and memory [13, 14]. In addition, osthole has been reported to have an inhibitory effect on multiple types of cancer, including breast cancer, cervical cancer, hepatic carcinomas, leukemia, and lung cancer [15–19]. The mechanism underlying these inhibitory effects is currently under investigation. Osthole's inhibition of the invasion of breast cancer cells *in vitro* has been demonstrated by research from several groups, including ours [15, 20]. Additionally, osthole has been implicated in the regulation of bone metabolism, and has been shown to have the ability to suppress bone loss and promote bone healing through controlling the differentiation of both osteoblasts and osteoclasts [21, 22]. Together, these results suggest the potential for osthole as a therapeutic candidate for inhibiting *in vivo* bone metastasis. In this study, we utilized a mouse model to investigate whether or not osthole could inhibit the metastasis of human breast cancer cells to bone.

## RESULTS

### Osthole inhibited bone metastasis in mice

**Figure 1:**
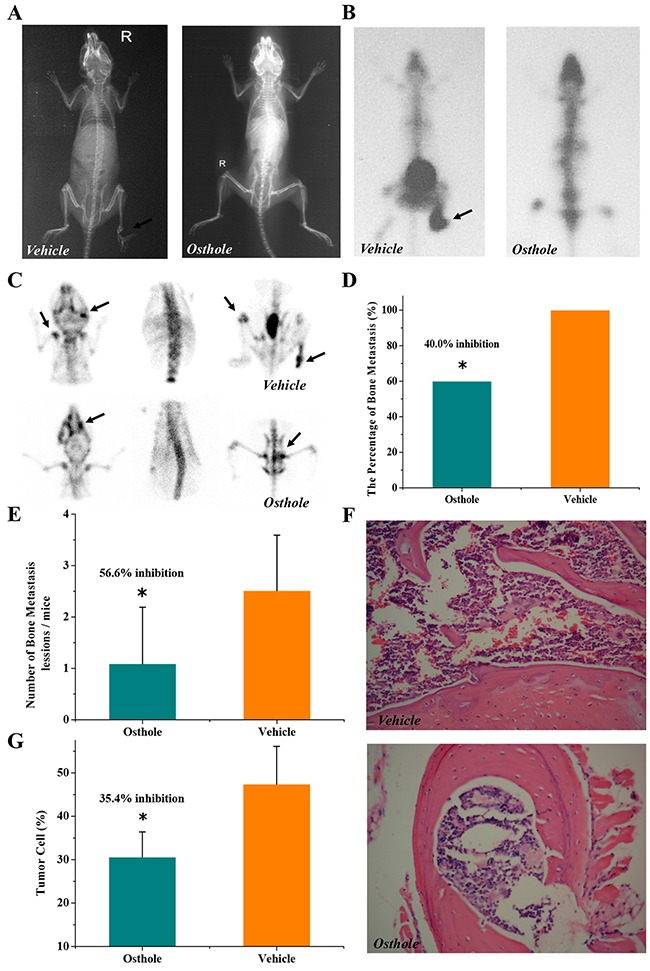
Osthole-mediated inhibition of breast cancer bone metastasis in mice **(A)** Radiograph images. **(B)** Whole-body bone scintigraphy images. **(C)** Pinhole bone scintigraphy images (arrows indicate sites of bone metastases). **(D)** Osthole diminished the incidence of bone metastasis (* *p* < 0.05 by the Fisher exact test). **(E)** Osthole reduced bone metastasis lesions (* *p* < 0.05 by ANOVA). **(F)** Representative images of osseous metastases. **(G)** Quantitative histomorphometry measurement of tumor burden in bone lesions (* *p* < 0.05 by ANOVA). Mice received an intracardiac injection of MDA-231BO cells, resulting in osseous metastases as detected by radionuclide bone scintigraphy and X-ray imaging within 2 weeks of tumor inoculation. The mice were then randomly divided into 2 groups (n=10 per group) that received either oral osthole (5.25 mg/kg) or vehicle twice weekly for 6 weeks. Every two weeks following inoculation, bone metastasis was evaluated by *in vivo* imaging with radiographs and radionuclide bone scintigraphy. Mice were sacrificed at the end of treatment, and metastatic bone lesions, observed by radionuclide scintigraphy and radiography, were sectioned and sent for H&E staining (magnification: 200X).

Mice with osseous metastases were divided randomly into 2 groups. To determine if osthole treatment could reduce osseous metastases, one group of mice was treated orally twice weekly with osthole (5.25 mg/kg), and the other group was treated identically with vehicle. After six weeks of treatment with osthole, the tumor metastasis rate to bone was suppressed by 40% on average and the number of metastatic lesions was reduced by approximately 57% when compared to vehicle-treated mice (Figure 1A–1E). We then evaluated the bones for metastatic lesions histologically and calculated the number of tumor cells present in the lesions. Osthole administration resulted in a significant reduction in tumor infiltration and an average 35% decrease in the percentage of tumor cells in metastatic lesions, when compared to vehicle-treated mice (Figure 1F/1G).

### Osthole regulated genes for bone metastasis and metabolism in mice

**Figure 2:**
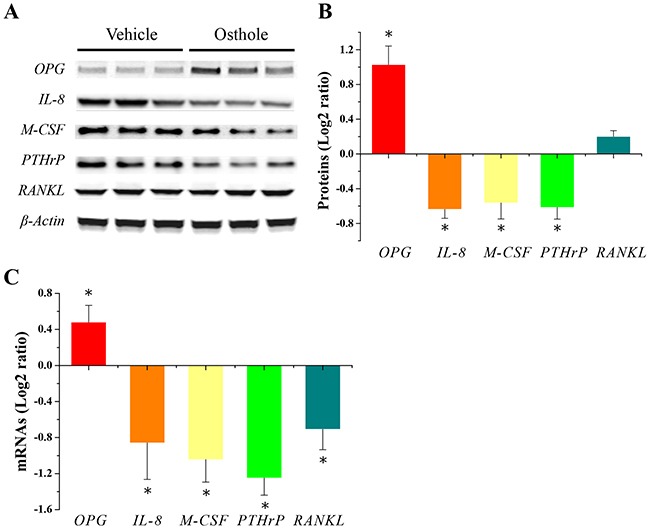
Alteration of bone metastatic and / or bone-related gene expression in osseous metastases **(A)** Representative images of protein expression by western blotting (n = 3 for each group). **(B)** Quantitative results of protein expression were made by two image analysis experts using Image J software (National Institutes of Health, Bethesda, MD, USA) (log2-ratio vs. vehicle; mean ± SD). * p < 0.05 vs. vehicle. **(C)** Quantitative results of mRNA expression determined with RT-qPCR. RT-qPCR measurements (log2-ratio vs. vehicle; mean ± SD) in 6 individual mRNA samples obtained from bone metastasis lesions with or without osthole treatment. * p < 0.05 vs. vehicle. Mice were sacrificed at the end of treatment, and metastatic bone lesions were harvested and stored in liquid nitrogen. Frozen tissue was homogenized with a pestle and total RNA and protein were extracted for RT-qPCR and western blotting, respectively.

In order to examine the means by which osthole treatment reduced metastatic growth, we harvested metastatic bone lesions and analyzed the gene products associated with bone metastasis and metabolism using real-time quantitative PCR (RT-qPCR) and western blotting. The resulting data revealed that, in osseous metastatic lesions, osthole significantly increased osteoprotegerin (OPG) and reduced interleukin-8 (IL-8), macrophage colony-stimulating factor (M-CSF) and parathyroid hormone-related peptide (PTHrP) protein expression (Figure 2A/2B). Interestingly, we found that the expression of bone-related mRNAs were similar to those proteins (Figure 2C). Together, our data suggest that the inhibitory effect of osthole on breast cancer bone metastasis may be due to the promotion of OPG and the inhibition of IL-8, M-CSF and PTHrP gene expression in tumor-bearing mice.

### Osthole suppressed cell viability and proliferation of MDA-231BO cells

**Figure 3:**
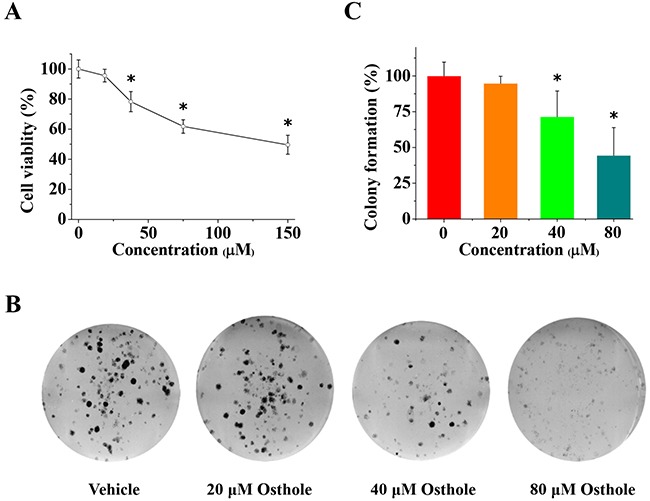
Osthole inhibited breast cancer cell viability and proliferation **(A)** Osthole treatment inhibited MDA-231BO cell viability. Cells were treated with vehicle or osthole (18.75, 37.5, 75 and 150 μM) for 24 h and then measured using MTT assays. Results are expressed as mean ± SD for three experiments (* *p* < 0.05 by ANOVA). **(B)** Representative images from colony formation assays using cells treated with either osthole or vehicle were taken at 24 h. **(C)** Quantitative results of colony formation (% of vehicle) expressed as the mean ± SD of three experiments. * p < 0.05 vs. vehicle. Colony formation of vehicle-treated cells was set at 100 %, and values for colony formation of osthole-treated cells were represented as a percentage of vehicle colony formation (* *p* < 0.05 by ANOVA).

We found that osthole significantly reduced tumor infiltration in metastatic lesions *in vivo*. In addition, osthole significantly reduced proliferation of human breast cancer MDA-MB 435 cells, but did not inhibit MCF-7 cell proliferation [15, 23]. To determine whether or not the reduction in osseous metastases was the result of diminished cellular viability, we used MTT assays to measure the effects of osthole on MDA-231BO cell viability. As shown in Figure 3A, MDA-231BO cells treated for 24 h with osthole at a concentration greater than or equal to 40 μM displayed significant inhibition in cellular viability *in vitro*. To further examine the effect of osthole on MDA-231BO cell proliferation, we carried out colony formation assay to evaluate its antiproliferative effects. As shown in Figure 3B/3C, concentrations of osthole greater than or equal to 40 μM significantly inhibited the proliferation of breast cancer cells *in vitro*. The disparity between previous study and our current results could be due to differences among the cell lines examined [see Supplementary Materials]. cell proliferation and reduces cell viability in human breast cancer cell bone-seeking subclone MDA-231BO.

### Osthole impaired the migration and invasion of MDA-231BO cells

**Figure 4:**
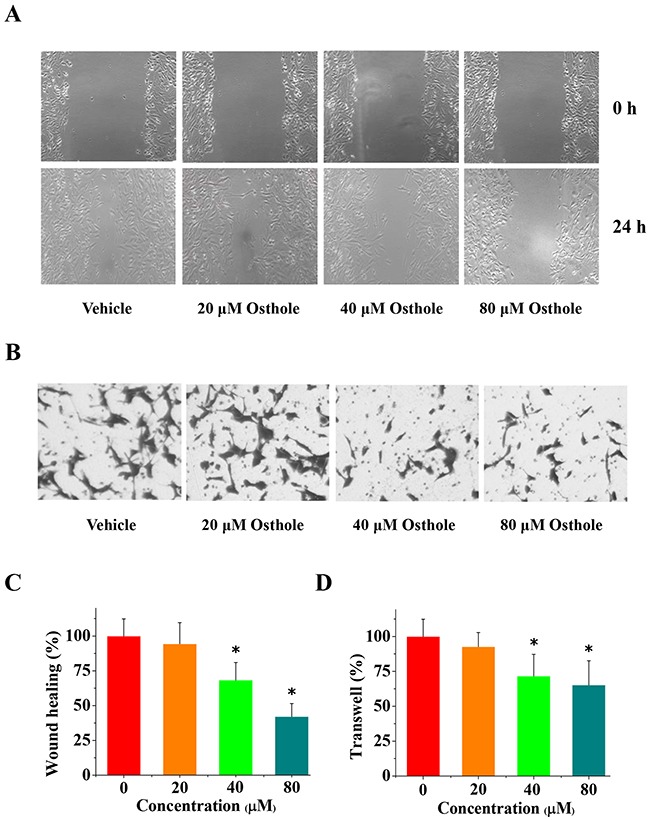
The anti-invasion and anti-migration effects of osthole on breast cancer cells **(A)** Representative images from wound healing assays using cells treated with either osthole or vehicle were taken at 0 h and 24 h. **(B)** Representative images of transwell analysis of cells treated with either osthole or vehicle were taken at 24 h. **(C)** Wound closure was quantified as the percentage of wound closure, and expressed as the mean ± SD of three experiments. Wound closure of vehicle-treated cells was set at 100 %, and wound closure of osthole-treated cells was represented as a percentage of the vehicle wound closure (* *p* < 0.05 by ANOVA). **(D)** Quantitative results of transwell migration assay (% of vehicle) expressed as the mean ± SD of three experiments. * p < 0.05 vs. vehicle. Transwell assay data, with vehicle-treated cells set at 100%, and osthole-treated cells represented as a percentage of the vehicle group (* *p* < 0.05 by ANOVA).

As described previously, osthole significantly reduced ER-positive breast cancer cell line MCF-7 migration [15]. Despite this, it is unclear whether osthole could inhibit cell migration in ER-negative breast cancer cell line MDA-231BO or not. We determine the role of osthole in cellular migration. MDA-231BO cells were treated with osthole for 24 h. Following treatment, the ability of MDA-231BO cells to migrate into wounds created by scratching confluent cells with a pipette tip was measured. As shown in Figure 4A/4C, MDA-231BO cells treated with osthole at the indicated concentrations displayed significant delays in wound closure resulting from diminished cellular migration. Quantification of wound size revealed that treatment of cells with osthole at a concentration of 80 μM inhibited wound closure by an average of 60 % as compared with vehicle-treated cells. Additionally, we assessed the inhibition of osthole on cell invasion using the transwell assay. The resulting data showed that osthole at concentrations greater than or equal to 40 μM significantly reduced cell invasion (Figure 4B/4D).

### Osthole induced apoptosis in MDA-231BO cells

**Figure 5:**
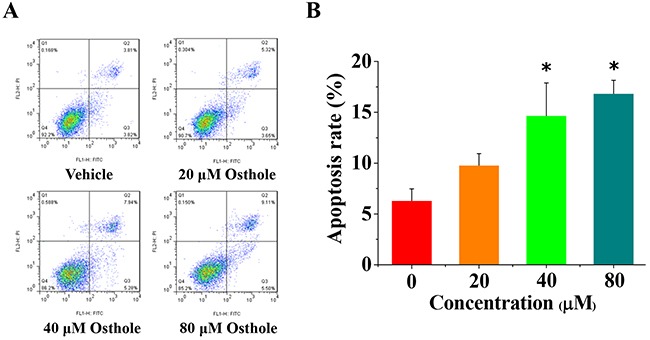
Osthole induced breast cancer cells apoptosis **(A)** Representative flow cytometry analysis data from Annexin V-FITC/PI staining. Cell apoptosis levels from cells treated with either osthole or vehicle were assessed at 24 h. **(B)** Quantitative results of cell apoptosis determined using Annexin V-FITC/PI flow cytometry analysis and expressed as the mean ± SD of three experiments. * p < 0.05 vs. vehicle.

Previously study shown that osthole induced cell apoptosis in breast cancer cell line MDA-MB 435 [23]. To confirm osthole's role in the regulation of cell apoptosis in MDA-231BO cells, we performed flow cytometric analyses specific for apoptosis. Consist with previous studies, treatment with osthole for 24 h significantly induced cell apoptosis in MDA-231BO cells when the concentrations of osthole were greater than or equal to 40 μM (Figure 5). Together, these data suggest that osthole induces apoptosis in MDA-231BO cells.

### Osthole inhibited the TGF-β/Smads signaling pathway in MDA-231BO cells

**Figure 6:**
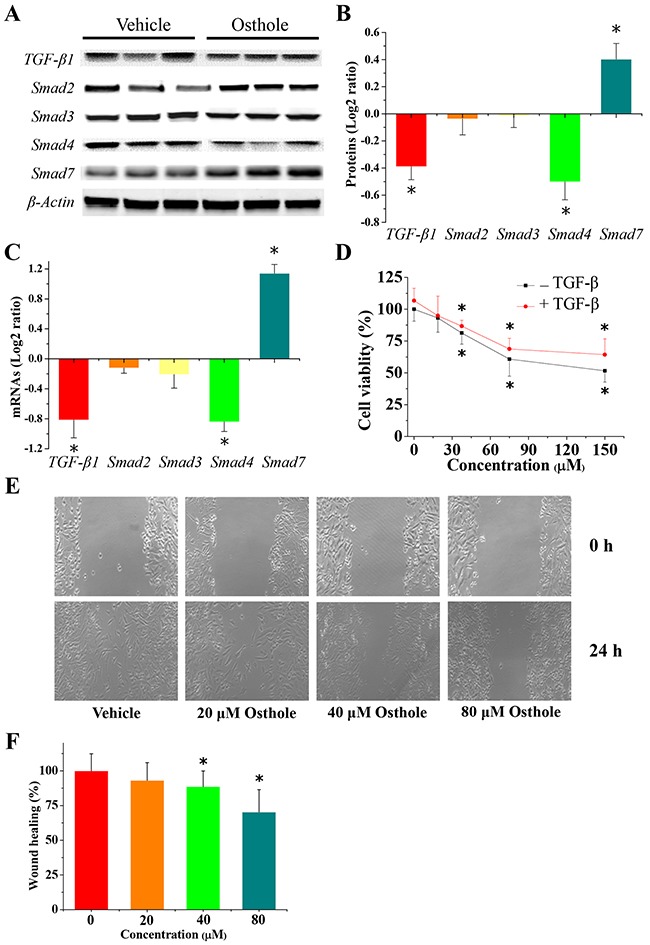
Alteration in gene expression of TGF-β/Smads signal pathway **(A)** Representative images of protein expression detected by western blotting. Cells were treated with or without 80 μM osthole for 24 h. Protein lysates were separated by SDS-PAGE and subjected to western blotting analysis using the specific antibodies indicated. **(B)** Quantification of the results of the protein expression (log2-ratio vs. vehicle; mean ± SD). Scanning densitometry was used for semiquantitative analysis for comparison to the vehicle-treated group. Results are expressed as the mean ± SD from three experiments. * p < 0.05 vs. vehicle. **(C)** Osthole-regulated TGF-β and Smad family gene expression. Cells were treated with or without 80 μM osthole for 24 h. Cells were collected and total RNA was extracted and analyzed using RT-qPCR. Measurements for RT-qPCR (log2-ratio vs. vehicle; mean ± SD) in 6 individual mRNA samples obtained from MDA-231BO cells treated with or without osthole. * p < 0.05 vs. vehicle. **(D)** Osthole treatment inhibited MDA-231BO cell viability when pretreated with TGF-β1. Cells were pretreated with 2 ng/mL TGF-β1 for 3 h, then were treated with indicated concentration of osthole for 24 h, and finally were measured by MTT assays. Results are expressed as mean ± SD for three experiments (* *p* < 0.05 by ANOVA). **(E)** Representative images from wound healing assays using cells treated with either osthole or vehicle were taken at 0 h and 24 h, when pretreated with TGF-β1. Cells were pretreated with 2 ng/mL TGF-β1 for 3 h, and then incubated with or without the indicated concentrations of osthole. After 24 h treatment with or without osthole, wound healing assays were begun, and representative scrape lines were imaged. **(F)** Wound closure was quantified as percentage of the wound closure from the control group, and expressed as the mean ± SD of three experiments. (* *p* < 0.05 by ANOVA).

Since TGF-β/Smad family signaling is involved in the malignant progression of breast cancer, we set out to investigate the gene expression of these proteins in MDA-231BO cells as well as the effect of osthole on their expression. We found that osthole significantly decreased the expression of TGF-β1 and Smad 4, and increased Smad 7 expression in MDA-231BO cells (Figure 6A–6C). We then further examined the regulation of osthole on TGF-β-induced cell viability and migration. Cells were pretreated with 2 ng/mL TGF-β1 for 3 h, and then incubated with the indicated concentrations of osthole. We initiated the MTT and wound healing assay after 24 h of treatment with or without osthole, and imaged the representative scrape lines. Although TGF-β actually promotes the proliferation and / or migration of cancer breast cancer cells, the effects of TGF-β on breast cancer line MDA-MB-231 and its subclone MDA-231BO are different with respect to other cell lines [24]. Additionally, TGF-β profoundly inhibited MDA-MB-231 cell growth, but did not have any inhibitory or proliferative effects on MDA-231BO cell growth [24]. Consistent with previous studies, we found that TGF-β did not promote MDA-231BO cell viability and cell migration (Figures 4A, 6D/6E). Ironically, our data suggested that osthole could inhibit wound healing in breast cancer cell line MDA-231BO pre-treated with TGF-β (Figure 6E/6F). Taken together, osthole probably regulated TGF-β/Smads signaling to inhibit breast cancer metastasis to bone.

## DISCUSSION

In the current study, osthole treatment resulted in a significant decrease in both the proliferation and migration of MDA-231BO cells *in vitro*. These results were consistent with previous reports demonstrating the antimigratory effects of osthole on cell lines *in vitro* [15]. In addition, we also found that osthole induced apoptosis in breast cancer cell MDA-231BO. Furthermore, we demonstrated that mice treated with osthole exhibited a significant reduction in tumor infiltration as well as a decrease in the percentage of tumor cells in bone metastasis lesions. Moreover, in tumor-bearing mice, osthole treatment resulted in a significant reduction in breast cancer cells metastasis to bone.

In previous studies, MCF-7 cells treated with osthole exhibited significantly reduced migration [15]. Interestingly, our current results indicated that osthole treatment also inhibited the migration of MDA-231BO cells. To explore whether or not the observed decrease in migration in response to osthole treatment was a consequence of reduced cellular viability and proliferation, we used MTT and colony formation assays. We found that, osthole significantly reduced the viability of MDA-231BO cells and inhibited cell proliferation. The results of wound healing and transwell analyses also showed that osthole significantly decreased cell migration and invasion. In addition, we observed that osthole significantly decreased tumor infiltration and reduced cancer cell proliferation in tumor-bearing mice. Furthermore, we found that osthole also induced apoptosis in MDA-231BO cells. Considering these results along with those from previous studies [15, 20, 23], we believe that osthole's inhibition of breast cancer cells *in vitro* and *in vivo* may depend, not only on antiproliferative effects, but also on antimigratory and apoptosis-inducing effects.

Bone homeostasis is a highly regulated balance between osteoblastic bone formation and osteoclastic bone resorption. Many molecular mediators have been implicated in this balance, such as OPG, RANK, RANKL, PTHrP, M-CSF and IL-8 [25]. Previous studies have demonstrated that osthole suppresses bone loss and promotes bone healing though regulating the differentiation of osteoblasts and osteoclasts [21, 22]. In our present study, osthole increased the expression of OPG and decreased the expression of IL-8, M-CSF, PTHrP, and RANKL in osseous metastases. Recently, Zhai *et al* showed that osthole enhances bone formation and inhibits bone resorption [26]. The authors verified that osthole decreases RANKL mRNA expression and stimulates mRNA expression of OPG in rat calvarial osteoblasts and bone marrow stromal cells. In addition, they also showed that osthole inhibits osteoclastic bone resorption via the regulation of TRAP activity. Taken together, these data and previous studies [21, 22, 26] support the hypothesis that osthole inhibits osteoblastic RANKL expression as well as differentiation of osteoclast precursors to osteoclasts *in vitro*. Collectively, these results indicated that osthole inhibits interactions among cancer cells, osteoblasts, and osteoclasts in osseous lesions.

The TGF-β/Smad family pathway plays a critical in breast cancer bone metastasis [27, 28]. The TGF-β released from the bone matrix as a result of increased bone resorption can act on tumor cells. This interaction causes the tumor cells to produce factors such as PTHrP and IL-11, resulting in further osteoclastogenesis and perpetuation of osteolytic disease [29]. In the present study, we found that osthole altered TGF-β and Smad levels in breast cancer MDA-231BO cells. We then examined the inhibitory effect of osthole on TGF-β-induced cell viability and migration. We found that, in MDA-231BO cells, osthole significantly inhibited cell viability and migration when pro-treated with or without TGF-β1. Interestingly, these results supported the hypothesis that osthole and its derivatives can effectively reduce dysfunctional TGF-β/Smads signaling [30]. These findings, backed by previous studies [20, 30], indicated that osthole regulates TGF-β signaling in breast cancer metastasis to bone in MDA-231BO cells.

**Figure 7:**
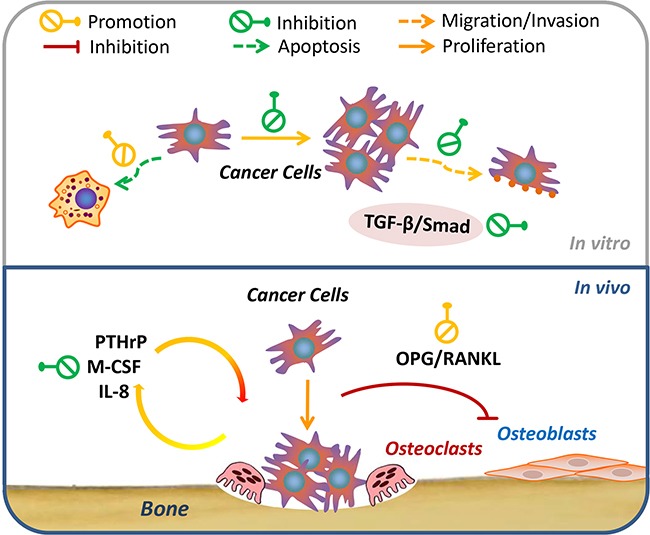
Schematic representation of the signaling pathways probably involved in osthole-mediated breast cancer bone metastasis Osthole inhibited breast cancer cell growth, migration, and invasion, and induced apoptosis of breast cancer cells *in vitro*. Additionally, it probably regulated TGF-β/SMADs signaling in the metastasis of breast to bone cancer in MDA-231BO cells. Besides, it also altered pro-metastatic / bone metabolic genes and regulated OPG/RANKL signals in the interactions between bone cells (osteoblasts and osteoclasts) and cancer cells *in vivo*. The green and yellow symbols indicate the inhibitory and promoting effects of osthole, respectively. Abbreviations: IL-8, interleukin-8; M-CSF, macrophage colony-stimulating factor; OPG, osteoprotegerin; PTHrP, parathyroid hormone-related peptide; RANKL, receptor activator of nuclear factor kappa-B ligand; TGF-β, transforming growth factor-beta.

Our results demonstrated that osthole significantly reduced bone metastasis and decreased the total tumor burden of breast cancer metastases in mice. In combination with previous work [15, 20–23, 26, 30], these findings suggest that osthole may potentially act on advanced breast cancer cells through the regulation of cellular viability, proliferation, migration, invasion, and apoptosis as well as through the interactions of osteoblasts, osteoclasts, and cancer cells during breast cancer bone metastasis (Figure 7). Therefore, osthole might have potential as a viable therapeutic candidate for the treatment of breast cancer patients with bone metastases.

## MATERIALS AND METHODS

### Materials

Osthole (purity >98%) was purchased from the National Institutes for Food and Drug Control, China. L-15 media and fetal bovine serum were obtained from Gibco (Grand Island, New York, USA). Antibodies against osteoprotegerin (OPG), receptor activator of nuclear factor kappa-B ligand (RANKL), macrophage colony-stimulating factor (M-CSF), parathyroid hormone-related peptide (PTHrP) and transforming growth factor-beta 1 (TGF-β1) were all obtained from Santa Cruz Biotechnology Biotechnology (Santa Cruz, CA). The antibody against interleukin-8 (IL-8) was purchased from Abcam Technology (Cambridge, MA). Smad 2, Smad 3 and Smad 4 antibodies were obtained from Cell Signaling Technology (Danvers, MA, USA). Smad 7 antibody was purchased from R&D Systems (USA). Nitro blue tetrazolium (NBT) and the 5-bromo-4-chloro-3-indolyl phosphate (BCIP) Color Development bone scintigraphy substrate were obtained from Promega Biotech (Madison, Wisconsin, USA). All other chemicals were purchased from Sigma-Aldrich (Saint Louis, Missouri, USA) unless otherwise indicated.

### Animals and experimental procedure

Female nude (BALB/c *nu/nu*) mice (20 ± 2 g; Shanghai Cancer Institute of Shanghai Jiaotong University) were housed in a temperature-controlled (24 ± 2°C) room with a regular 12-h light/dark cycle. After one week of acclimatization in a specific pathogen-free (SPF) environment, animals were randomly assigned to one of several experimental groups. All animals had free access to water and food until the day before the experiment. All animals had free access to food and water until the day prior to the experiment. All experiments were performed in accordance with national regulations for animal experimentation and approved by the Institutional Animal Care and Use Committee of the Shanghai Chest Hospital of Shanghai Jiaotong University (Permit Number: 20120219003).

Mice received an intracardiac injection of MDA-231BO cells, resulting in osseous metastases as detected by radionuclide bone scintigraphy and X-ray imaging within 2 weeks of tumor inoculation. The mice were then randomly divided into 2 groups (n=10 per group) that received either oral osthole (5.25 mg/kg) or vehicle twice weekly for 6 weeks. Every two weeks following inoculation, bone metastasis was evaluated by *in vivo* imaging with radiographs and radionuclide bone scintigraphy. Mice were sacrificed at the end of treatment, and metastatic bone lesions, observed by radionuclide scintigraphy and radiography, were sectioned and sent for H&E staining (magnification: 200X).

### Cell lines and cell culture

MDA-231BO cells were cultured in L-15 medium supplemented with 10% (v/v) fetal bovine serum at 37°C in a humidified atmosphere containing 5% CO_2_. Cells were washed three times and placed in sterile phosphate-buffered saline (PBS) shortly before implantation. An exclusive bone metastatic subclone, termed MDA-231BO, was acquired through repeatedly injecting MDA-231 breast cancer cells into the left ventricle and isolating tumor cells from bone metastasis lesions [31]. While the parental line (MDA-231) can develop metastases in bone, brain, ovary, and adrenal glands, MDA-231BO almost exclusively metastasizes to bone [24].

### Cell viability assay

Dose-response curves were determined using MTT assays. Cells (1 × 10^5^ cells/mL) were seeded in 96-well plates and incubated for 12 h. Osthole was diluted to the appropriate concentration and immediately added to the media. After 24 h of treatment, cell viability was evaluated by cell-mediated MTT reduction. Optical densities were detected at 570 nm.

### Colony formation assay

Cells were seeded in 6-well plates at a density of 500 cells per well, and after 10 days, the colonies with a diameter of >0.05 mm were counted.

### Wound healing assay

Cells were seeded in 6-well plates at a total of 8 × 10^4^ cells per well, and when cellular confluence reached about 90%, a 200 μl pipette tip was used to create wounds in the confluent cells. Wells were then rinsed with medium to remove any free-floating cells and debris, and media without serum containing various concentrations of osthole was added. Within 24 h after the scrape line was made, wound healing was observed and representative scrape lines were imaged. The percentage of wound area was calculated as follows: wound area (%) = (original wound area _treated_ - remaining wound area _treated_)/ original wound area _vehicle_ - remaining wound area _vehicle_) × 100. Each assay was conducted in triplicate.

### Transwell assay

MDA-231BO cells were seeded into transwell inserts at 5 × 10^4^ cells using 24-well transwell chambers (Corning) separated by a polycarbonate filter coated with 50 μg/ml collagen IV, with or without osthole at the indicated concentrations. After 24 h, cells on the top side of the inserts were scraped off and the transwell filters were stained with crystal violet and examined under an inverted microscope. The cells that had migrated within 24 h were quantified from triplicate wells.

### Apoptosis detection assay

A flow cytometric assay was performed to assess the effects of osthole on cell apoptosis as previously described [32]. Briefly, MDA-231BO cells were treated with osthole for 24 h, and then cells were harvested, and single-cell suspensions were prepared and stained with Annexin V-FITC/PI for apoptosis analysis. Cells were assessed using a Becton Dickinson FACScalibur machine and data were analyzed using CellQuest.

### Intracardiac injection

Intracardiac injections were performed as described previously [33]. Briefly, cells were resuspended at 10^6^ cells/mL in PBS. Suspended cells (0.1 mL) were injected into the left ventricle using 29 gauge needles (Terumo, Tokyo, Japan).

### Radionuclide bone scintigraphy

Radionuclide bone scintigraphy analyses were performed as described previously [33]. Following inoculation, bone metastasis was evaluated by *in vivo* imaging with radionuclide bone scintigraphy in 2-week intervals.

### Radiographic imaging

Radiographic imaging analyses were performed as described previously [33]. Briefly, conventional radiographs were obtained using a Philips Optimus Bucky Diagnost TS X-ray System (Philips Healthcare, Eindhoven, Netherlands). The X-ray tube voltage was fixed at 40 kVp, the current at 2 mA, and the exposure time at 3 s.

### Histology

After radionuclide scintigraphy and radiography analyses identified the bones with metastatic lesions, sample tissues were dissected, sectioned, and stained with hematoxylin and eosin (H&E). Histological evaluation of metastasis lesions was performed as described in our previous work [31]. Briefly, cancer cells were identified, and the percentage of cancer cells per high-power field-of-view (400× magnification) was calculated. All histological sections were examined by two independent pathologists. Sections that received substantially different scores from the two pathologists (>5% discrepancy) were reviewed again until a consensus was reached.

### Real-time quantitative PCR

Metastatic bone lesions were harvested and stored in liquid nitrogen. Frozen tissue (0.2–0.25 g) was homogenized with a pestle, and total RNA was extracted with TRIZOL reagent (Promega, Madison, WI) according to the manufacturer's instructions. Real-time PCR was performed as described in our previous work [33]. Primers were obtained from Shanghai Sangon Biological Engineering Technology & Services Co., Ltd. (Shanghai, China), and their sequences are shown in Table 1. The cycling conditions included an initial polymerase activation for 3 min at 94°C followed by 40 cycles of denaturation at 94°C for 30 s, annealing for 40 s at the temperatures indicated in Table 1, and elongation at 72°C for 30 s. RT-qPCR results for expression level were obtained using the delta delta CT method, by taking the threshold cycle number (CT) and, normalizing to the housekeeping gene, glyceraldehyde 3-phosphate dehydrogenase (GAPDH), then comparing relative to the vehicle. Finally, the values of each group were transformed to log2 ratio. P values were calculated using a *t*-test.

**Table 1:** Primer sequences used in real-time quantitative PCR analysis

Type	Name	Primer		Annealing temperature(°C)
		Type	Sequence, 5’-3’	
Mice	IL-8	Sense	ACA TGA CTT CCA AGC TGG CCG T	52
		Antisense	CCT CTT CAA AAA CTT CTC CAC AAC	
	M-CSF	Sense	AGC AGG AGT ATC ACC GAG GA	52
		Antisense	TAT CTC TGA AGC GCA TGG TG	
	PTHrP	Sense	ATG CAG CGG AGA CTG GTT CAG	58
		Antisense	TTC TAG TGC CAC TGC CCA TTG	
	OPG	Sense	CTT CGT GCC TTG ATG GA	44
		Antisense	TTG GGA AAG TGG GAT GT	
	RANKL	Sense	ACC AAG ATG GCT TCT ATT ACC	44
		Antisense	TCC CTC CTT TCA TCA GGT TAT	
	GAPDH	Sense	GGT CGG AGT CAA CGG ATT TG	58
		Antisense	ATG AGC CCC AGC CTT CTC CAT	
Cell	TGF-β1	Sense	AGC GAC TCG CCA GAG TGG TTA	58
		Antisense	GCA GTG TGT TAT CCC TGC TGT CA	
	Smad 2	Sense	TTA ACC GAA ATG CCA CGG TAG AA	60
		Antisense	GCT CTG GAC AAA CAT TGC ACT ATC A	
	Smad 3	Sense	AGG CGT GCG GCT CTA CTA CAT C	60
		Antisense	CAG CGA ACT CCT GGT TGT TGA A	
	Smad 4	Sense	CAG CAC TAC CAC CTG GAC TGG A	60
		Antisense	CTG GAA TGC AAG CTC ATT GTG AA	
	Smad 7	Sense	TGC TGT GCA AAG TGT TCA GGT G	60
		Antisense	CCA TCG GGT ATC TGG AGT AAG GA	
	GAPDH	Sense	GCA CCG TCA AGG CTG AGA AC	58
		Antisense	TGG TGA AGA CGC CAG TGG A	

### Western blotting

Frozen tissues (0.2–0.25 g) were homogenized using a pestle. Total protein was extracted and normalized, and western blot assays were performed as described previously with slight modifications [33]. Briefly, equal amounts of protein (20 μg) were separated by SDS-PAGE and transferred to nitrocellulose membranes. Membranes were blocked with 2% BSA and then incubated with the appropriate primary antibodies overnight at 4°C. Protein expression was detected by staining with NBT and BCIP. β-Actin was used as a loading control. Two imaging analysis experts have independently analyzed and quantified the immunoblotting data using Image J software (National Institutes of Health, Bethesda, MD, USA). The quantitative results generated by these two experts differed substantially (>5% discrepancy) and were further analyzed until an agreement was reached. The data were then normalized to the vehicle group. Finally, the values of each group were transformed to log2 ratio. P values were calculated using a *t*-test.

### Statistical analyses

All results are presented as mean ± standard deviation (SD). Two-tailed analysis of variance (ANOVA), followed by Dunnett's post hoc test and Fisher's test were used to determine the statistical significance. A *p* value < 0.05 was considered significant for all tests.

## SUPPLEMENTARY MATERIALS FIGURES AND TABLES



## Abbreviations

BCIP: 5-bromo-4-chloro-3-indolyl phosphate
*C. monnieri*: *Cnidium monnieri (L.) Cusson*
IL-8: interleukin-8
M-CSF: macrophage colony-stimulating factor
NBT: nitro blue tetrazolium
OPG: osteoprotegerin
PTHrP: parathyroid hormone-related peptide
RANKL: receptor activator of nuclear factor kappa-B ligand
RT-qPCR: real-time quantitative PCR
TGF-β: transforming growth factor-β
