# Supplementary material for: Osthole inhibits bone metastasis of breast cancer
Source: Oncotarget. 2017 Apr 11;8(35):58480–93. doi: 10.18632/oncotarget.17024 (PMC5601668; doi:10.18632/oncotarget.17024)
Supplement: Supplementary file 1 [file oncotarget-08-58480-s001.pdf]

Osthole inhibits bone metastasis of breast cancer

SUPPLEMENTARY MATERIALS

It is generally known that breast cancer metastatic sites are different due to variation in types of breast cancer cells. Past work has shown that the breast cell type MCF-7 is an estrogen receptor (ER) positive cell type that frequently metastasizes to lungs, bone, brain, and other tissue types (Sflomos *et al.* Cancer Cell 2016). In addition, MDA-MB-231 is an estrogen receptor (ER) negative cell type that usually metastasizes to bone, lungs, brain, ovary, adrenal, and other areas (Wang *et al.* Cancer Cell 2015; Yoneda *et al.* Journal of Bone & Mineral Research 2001). MDA-MB-231BO (also known as MDA-231BO) is a subclone of MDA-MB-231. It is possible to derive this subclone by repeatedly injecting MDA-MB-231 cells into the left ventricle and isolating the resulting tumor cells that have exclusively metastasized to bone (Yoneda *et al.* Journal of Bone & Mineral Research 2001). Based on the studies mentioned above, MDA-231BO was shown to be the most suitable cell line to study breast cancer that had exclusively metastasized to bone. This line was preferred relative to either its parental line (MDA-MB-231) or MCF-7.

Additional work by Yoneda *et al.* featured an *in vitro* comparison between MDA-MB-231 and its subclone MDA-231BO. Briefly, MDA-231BO produced greater amounts of parathyroid hormone-related protein (PTH-rP) than its parental line (MDA-MB-231). This difference in production was seen both in the absence and presence of transforming growth factor beta (TGF-β). Importantly, TGF-β profoundly inhibited MDA-MB-231 cell growth, but did not have any inhibitory or proliferative effects on MDA-231BO cell growth. The responses of MDA-MB-231 and MDA-231BO to TGF-β were quite different when compared with others breast cancer cells. Yoneda *et al*. took these findings to indicate that these phenotypic changes allowed breast cancer cells to promote osteoclastic bone resorption, survival, and proliferation in bone. Consequently, these effects would lead to the establishment of bone metastases. Given these data, we chose MDA-231BO to study the inhibitory effects of osthole on how breast cancer metastasizes to bone cancer.

Yang *et al*. used MCF-7 cells and found that osthole did not inhibit the growth of breast cancer (Yang *et al.*, Bioscience, Biotechnology, and Biochemistry 2010). However, we found that osthole significantly inhibited the growth of breast cancer MDA-231BO cells in the current study. It is notable that this osthole-derived antiproliferative effect differed from that previously reported by Yang *et al.* (Yang *et al.*, Bioscience, Biotechnology, and Biochemistry 2010). However, Wang *et al.* also recently found that osthole inhibited proliferation in another human breast cancer cell line (MDA-MB 435) through the induction of cell cycle arrest and apoptosis (Wang *et al.* Journal of Biomedical Research 2015). Thus, the disparity between previous study and our current results could be due to differences among the cell lines examined. Furthermore, we have now briefly summarized the similarities and differences among three breast cancer cell lines in Tables 1-3 (see below).

REFERENCES

1. Wang H, Yu C, Gao X, Welte T, Muscarella AM, Tian L, Zhao H, Zhao Z, Du S, Tao J, Lee B, Westbrook TF, Wong ST, et al. The osteogenic niche promotes early-stage bone colonization of disseminated breast cancer cells. Cancer Cell. 2015; 27: 193-210.
2. Sflomos G, Dormoy V, Metsalu T, Jeitziner R, Battista L, Scabia V, Raffoul W, Delaloye JF, Treboux A, Fiche M, Vilo J, Ayyanan A, Brisken C. A preclinical model for ERα-positive breast cancer points to the epithelial microenvironment as determinant of luminal phenotype and hormone response. Cancer Cell. 2016; 29: 1-16.
3. Hiraga T, Ito S, Nakamura H. Cancer stem-like cell marker CD44 promotes bone metastases by enhancing tumorigenicity, cell motility, and hyaluronan production. Cancer Res. 2013; 73: 4112-22.
4. Yoneda T, Williams P J, Hiraga T, Niewolna M, Nishimura R. A bone-seeking clone exhibits different biological properties from the MDA-MB-231 parental human breast cancer cells and a brain-seeking clone *in vivo* and *in vitro*. J Bone Miner Res; 2001: 16:1486-95.

SUPPLEMENTARY TABLES

Supplementary Table 1: Characteristics of breast cancer cell lines MCF-7 and MDA-MB-231

Types	ER	Bone metastasis*	Tumor inoculation	References
MCF-7	+	42 day	Intrailiac artery injection	Wang *et al.* Cancer Cell 2015
MDA-MB-231	-	29 day	Intrailiac artery injection	Wang *et al.* Cancer Cell 2015
Types	ER	Metastasis sites	Tumor inoculation	
MCF-7	+	Lungs/ Brain	Mammary fat pad	Sflomos *et al.* Cancer Cell 2016
MDA-MB-231	-	Lungs/ Brain/ bone/ Ovary/ Adrenal	Intracardiac inoculation	Wang *et al.* Cancer Cell 2015; Hiraga *et al.* Cancer Research 2013; Yoneda *et al.* Journal of Bone & Mineral Research 2001

* Ten-fold signal intensity at the initial injection day.

Supplementary Table 2: Characteristics of MDA-231BO and its parental cell line MDA-MB-231*

Types	Mice with metastasis		Tumor inoculation	References
	Bone	Nonbone		
MDA-MB-231	100% (7/7)	57% (4/7)	Intracardiac inoculation	Yoneda *et al.* Journal of Bone & Mineral Research 2001
MDA-231BO	100% (8/8)	0% (0/8)	Intracardiac inoculation	

* Yoneda *et al*. Journal of Bone & Mineral Research 2001

Supplementary Table 3: Characteristics of MDA-231BO and its parental MDA-MB-231*

Characteristics	**MDA-MB-231**	**MDA-231BO**
Site of passage	-	Bone
Tumorigenicity at orthotopic site	Yes	Yes
Site of metastasis	Bone, Brain, Ovary, Adrenal	Bone
PTH-rP production	Low	High
Resistance to growth-inhibitory effect of TGF-β	No	Yes
Growth-inhibitory effect of TGF-β	Yes	No
Growth-promotion effect of TGF-β	No	No
Growth-promotion stimulation by IGF	No	Yes
Growth-inhibitory stimulation by IGF	No	No

* Yoneda *et al*. Journal of Bone & Mineral Research 2001
